# Chikungunya Virus Replication in Salivary Glands of the Mosquito *Aedes albopictus*

**DOI:** 10.3390/v7112917

**Published:** 2015-11-17

**Authors:** Anubis Vega-Rúa, Christine Schmitt, Isabelle Bonne, Jacomine Krijnse Locker, Anna-Bella Failloux

**Affiliations:** 1 Arboviruses and Insect Vectors Unit, Department of Virology, Institute Pasteur, 25-28 rue du Docteur Roux, 75724 Paris cedex 15, France; anubis.vega-rua@pasteur.fr (A.V.-R.); 2 Ultrapole, Center for Innovation and Technology Research, Institute Pasteur, 25-28 rue du Docteur Roux, 75724 Paris cedex 15, France; christine.schmitt@pasteur.fr (C.S.); isabelle.bonne@pasteur.fr (I.B.); jacomina.krijnse-locker@pasteur.fr (J.K.L.)

**Keywords:** Chikungunya virus, *Aedes albopictus*, salivary glands, budding, replication, transmission electron microscopy

## Abstract

Chikungunya virus (CHIKV) is an emerging arbovirus transmitted to humans by mosquitoes such as *Aedes albopictus*. To be transmitted, CHIKV must replicate in the mosquito midgut, then disseminate in the hemocele and infect the salivary glands before being released in saliva. We have developed a standardized protocol to visualize viral particles in the mosquito salivary glands using transmission electron microscopy. Here we provide direct evidence for CHIKV replication and storage in *Ae. albopictus* salivary glands.

## 1. Introduction

**Figure 1:**
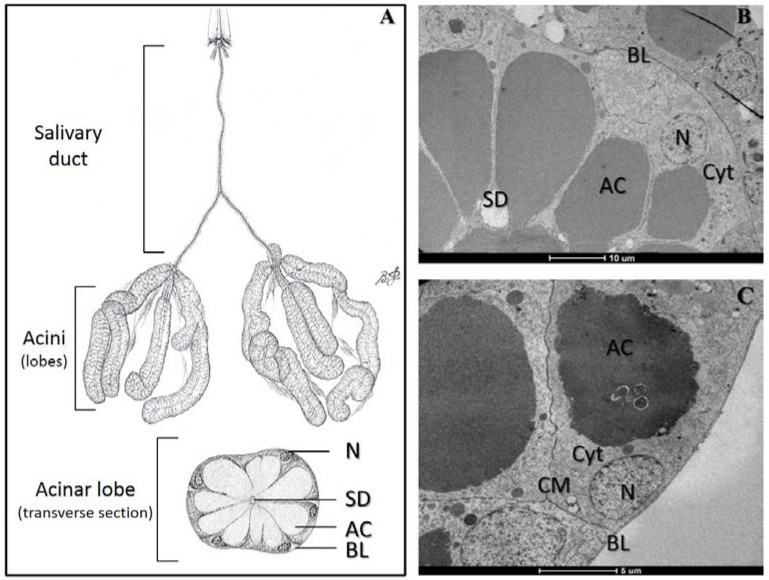
Structure of *Ae. albopictus* salivary glands. (**A**) Schematic representation of salivary glands in *Aedinae* mosquitoes (Modified from Jobling, 1987); (**B**,**C**) Ultrastructural views of transverse-sections of an acinar lobe or acinus. N: nuclei. SD: salivary duct. AC: apical cavity for saliva storage. BL: basal lamina. Cyt: Cytoplasm. CM: Cell membrane. Images were captured using a FEI Tecnai Biotwin transmission electron microscope (Eindhoven, The Netherlands).

Chikungunya fever (CHIK) is a severe and debilitating disease that often produces chronic arthralgia in humans [1]. This disease is caused by chikungunya virus (CHIKV), a globally emerging arbovirus transmitted to humans through infectious saliva delivered by mosquitoes when taking a blood meal [2,3,4]. More precisely, CHIKV is ingested by the mosquito during a blood meal on a viremic host. After penetrating the midgut cells, CHIKV must replicate and disseminate to the hemocele, subsequently infecting other organs such as the salivary glands [5,6,7]. Mosquito salivary glands are paired organs located in the thorax of the adult. Each gland is composed of three lobes (Figure 1), two lateral and one median. Each lobe consists of a basal lamina bounding a single layer of epithelial or acinar cells, which are distributed around a central salivary duct containing an apical cavity for saliva storage [5,6,7]. Therefore, to be transmitted, CHIKV present in the hemocele must (i) penetrate the basal lamina of the salivary glands surrounding acinar cells; (ii) replicate inside these cells; and (iii) be deposited into the apical cavities where mosquito saliva is stored prior to its release during feeding. As the route of CHIKV through the mosquito salivary glands is still poorly understood [7], we developed a protocol to visualize CHIKV in *Ae. albopictus* salivary glands using transmission electron microscopy (TEM) [8]. Furthermore, we provide direct evidence of CHIKV replication and storage in salivary glands of *Ae. albopictus*, a major CHIKV vector.

## 2. Results

**Figure 2:**
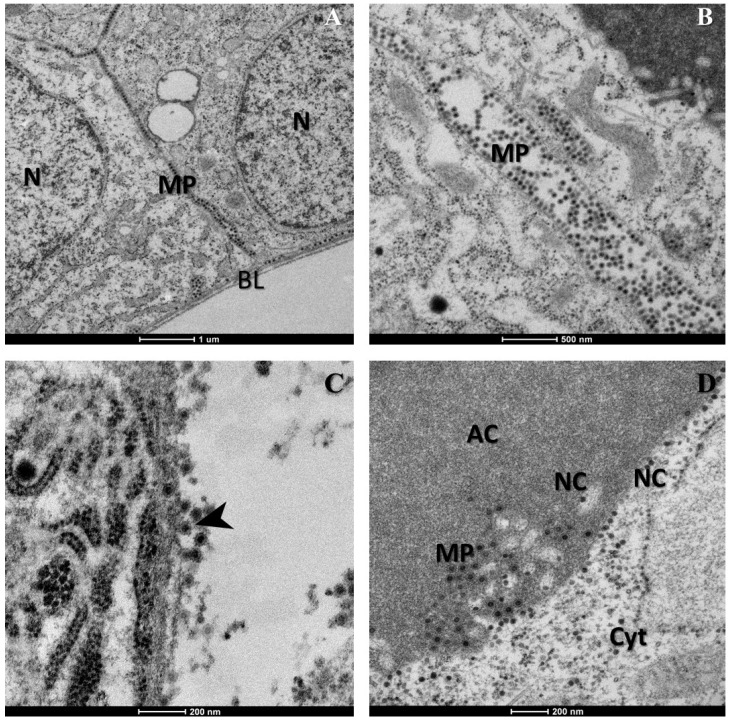
Salivary glands acinar cells infected with Chikungunya virus (CHIKV). (**A**,**B**) Mature CHIKV particles (MP) are seen in cell junctions. BL: basal lamina. N: nuclei; (**C**) Arrows point some budding virions at the plasma membrane (**D**) overview of an infected cell where nucleocapsids (NC) and mature virions (MP) are observed in cell junctions, cytoplasm and apical cavity (AC). Images were captured using a FEI Tecnai Biotwin transmission electron microscope.

**Figure 3:**
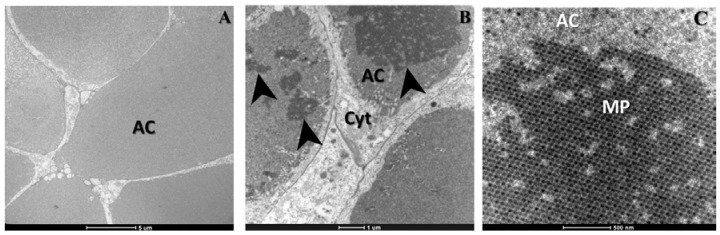
CHIKV stored in the apical cavities of salivary glands acinar cells of *Ae. albopictus*. (**A**) non-infected apical cavities; (**B**,**C**) apical cavities infected with CHIKV; (**B**) CHIK virions aggregations (black arrows); (**C**) detail (zoom) of one aggregation of CHIK mature virions (MP) whose envelope is clearly observed. AC: apical cavity. Cyt: cytoplasm. Images were captured using a FEI Tecnai Biotwin transmission electron microscope.

**Figure 4:**
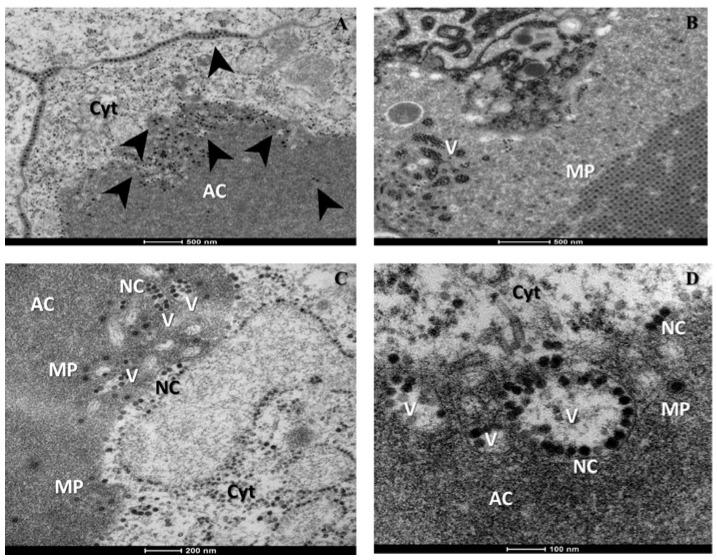
CHIKV entry in the apical cavities of salivary glands acinar cells of *Ae. albopictus*. (**A**) Overview of CHIKV in the acinar cell (black arrows); (**B**,**C**) detail of apical cavities (AC) where numerous vesicles (V) containing CHIKV nucleocapsids (NC) are shown. CHIKV mature particles (MP) are always observed outside the vesicles; (**D**) detail of vesicles containing CHIKV nucleocapsids in the apical cavity of the acinar cell. Cyt: cytoplasm. Images were captured using a FEI Tecnai Biotwin transmission electron microscope.

Figure 2 shows the acinar cells of salivary glands infected with CHIKV. In Figure 2A,B, we observed mature CHIKV particles ~60 nm in diameter composed of a central dense core surrounded by a viral envelope, located in cell junctions and near the basal lamina. The same features were observed in both experiments. No cytopathic effects were observed in cells, however evidence of viral replication was observed including: (i) CHIKV particles budding at the cell plasma membrane (Figure 2C) and (ii) the presence of several CHIKV nucleocapsids and mature particles inside these cells (Figure 2D). Nucleocapsids were visualized as dense cores without envelope of ~30 nm and were mainly found in the cell cytoplasm while mature CHIKV particles were generally found in cell junctions or in the apical cavities of cells (Figure 2D) where CHIK virions are deposited after replication [7,9]. Indeed, Figure 3B,C showed a remarkable arrangement of numerous mature CHIK virions in apical cavities of acinar cells, where the virus is stored along with saliva [5,7]. At the borders of these apical cavities CHIKV nucleocapsids were always observed inside vesicles (Figure 2D and Figure 4B–D), surrounded by mature CHIKV particles. This observation suggested that nucleocapsids might bud at these vesicle membranes, which should therefore contain processed CHIKV envelope glycoproteins, similar to the classical description of CHIKV budding through the cell plasma membrane [10,11,12]. Nevertheless, further studies should be conducted in order to corroborate this hypothesis as well as the possibility that the vesicles may be invaginations of the limiting membrane. Furthermore, we did not directly observe budding at the membrane of these vesicles.

## 3. Discussion

Our study provides direct evidence of CHIKV replication and storage in *Ae. albopictus* salivary glands. In this organ, CHIKV replication did not produce any remarkable cytopathic effect by day 6 post-infection. Nevertheless, it is possible that CHIKV causes some cellular damage in salivary glands after day 6 post-infection as it has it has been shown for Sindbis virus [13]. Our observations also contrast those of Soekiman and colleagues in 1987 [8]. While they did not see CHIKV budding, we have shown that in *Ae. albopictus* salivary glands CHIKV nucleocapsids seem to bud from two different sites within acinar cells to become mature particles: (i) at the cell plasma membrane as it has been traditionally described [10,11,12] and (ii) at the membrane of vesicles located in the apical cavity of acinar cells. This particular feature of the CHIKV life cycle may be related to the polarity of acinar cells and their secretory function [5,6,7]. Nevertheless, further studies are required to corroborate this observation and to better understand CHIKV trafficking in the salivary glands, specifically the mechanisms involved in salivary gland infection and escape barriers preventing infection and release of virus from the infected salivary glands, respectively [7,14]. Beyond increasing our understanding of CHIKV-vector interactions, the technical approach described here may contribute to the study of other medically important arboviruses and redefine the role of salivary glands in transmission.

## 4. Materials and Methods

### 4.1. Mosquito Rearing and Oral Infection

Lab reared adult female mosquitoes derived from *Ae. albopictus* collected in Manaus (Brazil) in 2013 were used in this study [15]. Mosquitoes were collected as eggs and after hatching, larvae were split into pans of 200 individuals, fed with 1 yeast tablet dissolved in 1 L of tap water and were both replaced every 48 h. Adults were maintained in cages at 28 °C ± 1 °C with a 16 h:8 h light:dark cycle, 80% relative humidity, and supplied with a 10% sucrose solution. CHIKV 06.21 strain was used for all infection assays. Belonging to the East-Central-South African lineage, CHIKV 06.21 was isolated in 2005 from a newborn male presenting meningo-encephalitis symptoms [16] and was kindly provided by the French National Reference Center for Arboviruses at the Institut Pasteur in Paris. CHIKV 06.21 strain harbors a valine at position 226 of the E1 envelope glycoprotein (E1-226V). This mutation has been shown to enhance *Ae. albopictus* vector competence for CHIKV [17,18]. The entire experiment was repeated twice. For each experiment, five to seven day-old adult female mosquitoes were fed an infectious blood-meal containing 1.4 mL of washed rabbit erythrocytes and 700 μL of viral suspension supplemented with a phagostimulant (ATP) at a final concentration of 5 mM. The titer of infectious blood-meals was 10^7.5^ PFU/mL [15]. After the infectious blood-meal, fully engorged mosquitoes were transferred to cardboard containers and maintained with 10% sucrose at 28 °C ± 1 °C, a 16 h:8 h light:dark cycle and 80% humidity.

### 4.2. Salivary Glands Dissection and Treatment for Transmission Electron Microscopy

At day 6 post-infection, salivary glands attached to the head were removed in PBS under a dissecting microscope. We chose this time point based on previous studies, where the proportion of *Ae. albopictus* mosquitoes with infected saliva and the number of CHIKV particles in saliva were shown to be higher 6–7 days after ingestion of CHIKV [15,19,20]. Salivary glands were transferred to transwell cell culture inserts (Corning) and fixed in 2.5% glutaraldehyde with 0.1 M cacodylate buffer (pH 7.2) O/N at +4 °C, then washed in 0.2 M cacodylate buffer (pH 7.2), post-fixed for 1 h in 1% osmium, and rinsed with distilled water. Salivary glands were immobilized in Agar type 9 diluted at 4% in water, then dehydrated through ascending ethanol bathes, infiltrated using Epon812 resin and baked 48 h at 60 °C. Samples were sliced in ultrathin 70 nm sections using a Leica UltraCut UC7 (Leica Microsystems, Vienna, Austria), stained using 4% uranyl acetate, and viewed on FEI Tecnai Biotwin transmission electron microscope (Eindhoven, The Netherlands) at 80 KV. Images were captured using Eagle 4K CCD (Eindhoven, The Netherlands) and TIA software (FEI^®^) (Eindhoven, The Netherlands).
